# Mechanisms underlying the association between perceived recurrence risk and negative emotions in patients with ischemic stroke: a cross-sectional study

**DOI:** 10.3389/fpsyt.2026.1865589

**Published:** 2026-07-22

**Authors:** Renlong Liang, Xuyan Liu, Rui Jian, Heng Ding, Tingting Cai

**Affiliations:** 1 Department of Neurology, Deyang Hospital Affiliated Hospital of Chengdu University of Traditional Chinese Medicine, Deyang, Sichuan, China; 2 Department of Nursing, Deyang People’s Hospital, Deyang, Sichuan, China

**Keywords:** family care, help-seeking attitudes, ischemic stroke, moderated mediation, negative emotions, perceived recurrence risk

## Abstract

**Objective:**

To examine the relationships among perceived risk of recurrence, attitudes toward seeking professional psychological help, negative emotions, and family care in patients with ischemic stroke, and to test a moderated mediation model. The findings aim to provide a scientific basis for optimizing clinical psychological interventions and family support strategies.

**Methods:**

A cross-sectional survey was conducted among 311 patients with ischemic stroke recruited from three tertiary hospitals in Sichuan Province, China. Data were collected using validated scales measuring perceived recurrence risk, help-seeking attitudes, negative emotions, and family care. Mediation and moderated mediation analyses were performed using SPSS 27.0 and PROCESS macro (Models 4 and 58), with 5, 000 bootstrap resamples to estimate 95% confidence intervals (CIs).

**Results:**

Perceived recurrence risk was positively associated with negative emotions (*β = 0.350, p < 0.001*). Attitudes toward seeking professional psychological help partially mediated this association, with a significant indirect effect (*effect = 0.131, 95% CI: 0.082–0.192*), accounting for 40.68% of the total effect. Family care moderated both paths of the mediation model. In the first stage, family care moderated the association between perceived recurrence risk and help-seeking attitudes (*β = 0.043, p = 0.001*), such that higher family care weakened the negative association. In the second stage, family care moderated the association between help-seeking attitudes and negative emotions (*β = −0.075, p = 0.008*), indicating that higher family care strengthened the protective association between help-seeking attitudes and lower negative emotions.

**Conclusion:**

Perceived recurrence risk, help-seeking attitudes, and negative emotions are significantly associated in patients with ischemic stroke. Help-seeking attitudes partially explain this association, while family care plays a significant moderating role in both stages. These findings highlight the importance of psychological and family-centered support in stroke rehabilitation. However, causal interpretations should be made cautiously due to the cross-sectional design.

## 1 Introduction

Ischemic stroke, an acute cerebrovascular disease, is the most prevalent subtype of stroke worldwide, accounting for approximately 70%–80% of all stroke cases (1). Its high rates of disability and recurrence impose substantial social and economic burdens, making it a major public health concern globally, particularly in China. According to the China Stroke Surveillance Report 2021 (2), the 5-year recurrence rate of ischemic stroke in China is approximately 41%, with a 1-year recurrence rate of about 17%, which is comparable to global estimates. Notably, patients with recurrent stroke exhibit significantly greater neurological deficits and higher mortality rates than those experiencing a first-ever stroke.

With the growing adoption of the biopsychosocial medical model, psychological health issues in stroke patients have become a research priority. Negative emotions, primarily anxiety and depression, affect approximately 30%–50% of patients with ischemic stroke (3). Previous studies have suggested that perceived recurrence risk may indirectly influence emotional outcomes by affecting individuals’ psychological coping processes, and it is also associated with treatment adherence and quality of life (4). However, the underlying psychological mechanisms remain insufficiently clarified. It is important to note that the present study focuses on the pathways through which perceived recurrence risk affects negative emotions in patients with ischemic stroke. This focus aligns with the emerging trend of integrated physical–psychological interventions in global stroke rehabilitation research highlighting the importance of elucidating the internal mechanisms linking these key variables (5). Therefore, this study aims to examine the underlying pathways through which perceived recurrence risk influences negative emotions in patients with ischemic stroke, from the perspectives of psychological coping and social support, thereby providing theoretical evidence for clinical psychological interventions.

### 1.1 Perceived recurrence risk and negative emotions

Perceived recurrence risk refers to patients’ subjective perception of the likelihood of disease recurrence. Within the Stress and Coping Theory framework (4), it is considered an important cognitive stressor that influences psychological outcomes in individuals with chronic diseases. Previous studies have demonstrated a significant positive association between perceived recurrence risk and negative emotions in patients with ischemic stroke (6, 7). However, several limitations remain. First, most studies have focused on the direct relationship between these variables, while the underlying mechanisms through which perceived recurrence risk influences negative emotions remain insufficiently explored, and potentially modifiable psychological pathways are still unclear (8). Second, existing evidence is largely based on heterogeneous stroke populations, whereas research specifically targeting ischemic stroke—a subtype accounting for more than 80% of all stroke cases—remains limited, reducing the specificity and clinical applicability of existing findings (9). These gaps hinder the development of precision-based psychological interventions and are not fully aligned with current rehabilitation demands under the precision medicine framework.

### 1.2 The mediating role of help-seeking attitudes

Within the Stress and Coping Theory framework, the impact of stressors on emotional outcomes is primarily mediated through individuals’ cognitive appraisal and coping processes, rather than exerting a direct effect on emotions (10, 11). As a key stressor, perceived recurrence risk may influence psychological outcomes by shaping coping tendencies.

Help-seeking attitudes refer to individuals’ cognitive evaluations and behavioral tendencies toward seeking professional psychological support when experiencing psychological distress, representing a coping orientation rather than actual help-seeking behavior (12). Among patients with ischemic stroke, higher perceived recurrence risk is often accompanied by persistent feelings of disease threat and uncertainty (13). Such prolonged stress may trigger avoidant coping tendencies, including reduced willingness to utilize external psychological resources and a lower likelihood of actively seeking professional help, thereby limiting access to effective emotional regulation resources and increasing the risk of negative emotions (14).

Therefore, from the perspective of the “stress appraisal–coping tendency–emotional outcome” pathway, help-seeking attitudes may play a mediating role between perceived recurrence risk and negative emotions. However, empirical evidence validating this mechanism in ischemic stroke populations remains limited, and the specific pathways require further clarification.

### 1.3 The moderating role of family care

The buffering hypothesis within Social Support Theory (15). suggests that social support does not directly determine psychological outcomes but instead protects individuals by buffering the impact of stressors on psychological responses. When individuals face high levels of stress, adequate social support can alleviate psychological strain and reduce the likelihood of negative emotional outcomes (16).

As the most important source of social support for patients with ischemic stroke, family functioning plays a crucial role in determining both the level and effectiveness of support received (17). The Family APGAR Index assesses family functioning across multiple dimensions, including adaptation, partnership, growth, affection, and problem-solving, and serves as a validated measure of perceived family support quality (18).

Within this theoretical framework, family care may exert moderating effects through two mechanisms. First, higher levels of family care may provide stable emotional and instrumental support, thereby buffering the psychological impact of perceived recurrence risk and weakening its association with negative emotions (19). Second, well-functioning family systems may enhance patients’ overall psychological adaptability, reducing excessive emotional reactivity even in the presence of high perceived risk (20). Conversely, poor family functioning may result in insufficient support, increasing vulnerability to stressors and exacerbating negative emotional responses (21).

However, empirical studies examining the moderating role of family functioning, particularly using standardized instruments such as the APGAR scale, in the relationship between risk perception and emotional outcomes in ischemic stroke populations remain limited.

### 1.4 Research hypotheses

**Figure 1:**
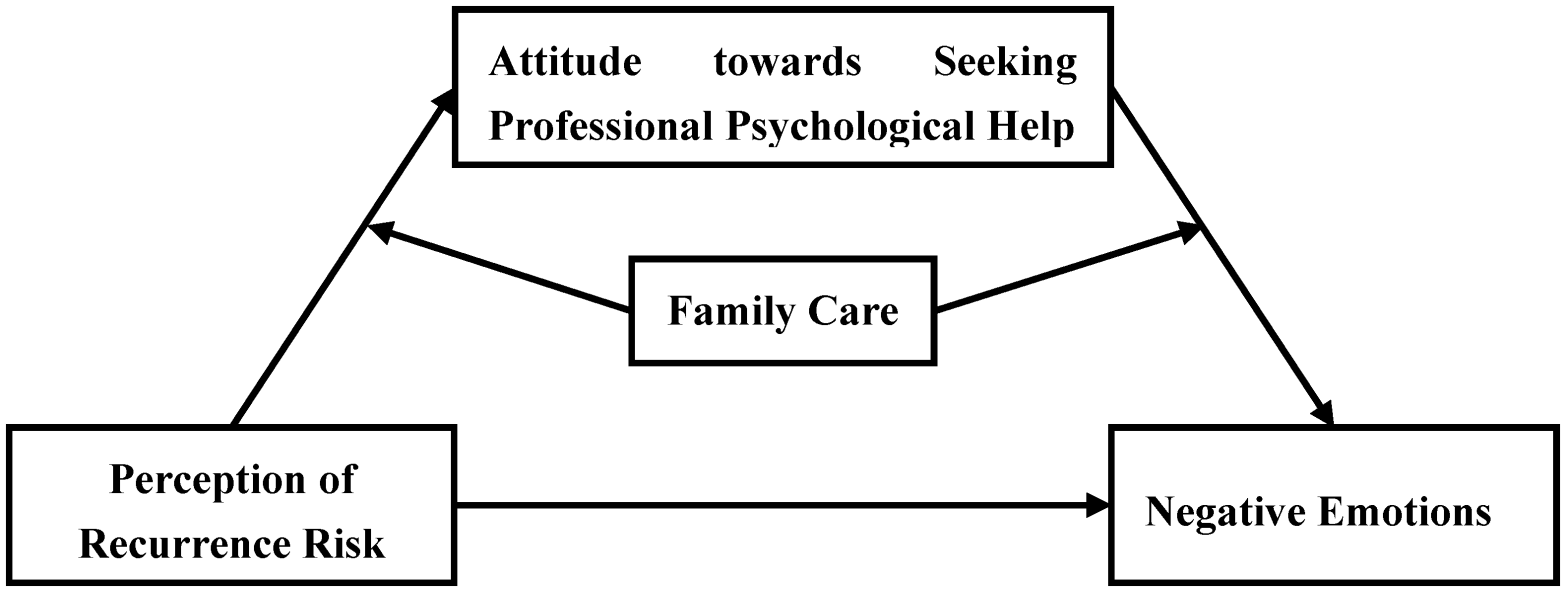
Hypothesis-based structural equation diagram.

Based on the above analysis and grounded in Stress and Coping Theory (4) and Social Support Theory (15), this study constructs a moderated mediation model in which attitudes toward seeking professional psychological help serve as a mediator and family care acts as a moderator (see **Figure 1**). This framework aligns with the emerging emphasis on proactive mental health promotion and family-centered rehabilitation in clinical practice. The study proposes the following hypotheses:

Hypothesis 1: Perceived recurrence risk was positively associated with negative emotions in patients with ischemic stroke.

Hypothesis 2: Help-seeking attitudes mediate the relationship between perceived recurrence risk and negative emotions.

Hypothesis 3: Family care moderates the relationship between perceived recurrence risk and help-seeking attitudes, such that the association is weaker at higher levels of family care.

Hypothesis 4: Family care moderates the relationship between help-seeking attitudes and negative emotions, such that the protective effect of help-seeking attitudes is stronger at higher levels of family care.

## 2 Materials and methods

### 2.1 Study design

This study employed a cross-sectional survey design. Participants were recruited using a convenience sampling method from three tertiary hospitals in Sichuan Province, China, between April 2025 and March 2026. Data were collected using structured questionnaires, including sociodemographic and clinical characteristics, the Perceived Recurrence Risk Scale, the Attitudes Toward Seeking Professional Psychological Help Scale, a Negative Emotion Scale, and the Family APGAR Index. This study strictly adhered to the principles of the Declaration of Helsinki. Written informed consent was obtained from all participants prior to data collection.

### 2.2 Sample size calculation

An *a priori* power analysis was performed using GPower 3.1 (22) for linear multiple regression with a medium effect size (f² = 0.15), α = 0.05, power = 0.95, and 14 predictors, yielding a minimum required sample size of 226 participants. To allow for incomplete or invalid questionnaires, the target sample size was increased to 249 participants. A total of 311 eligible participants were ultimately included. Because GPower does not estimate statistical power for moderated mediation models, an additional Monte Carlo simulation–based power analysis was conducted using the observed path coefficients from the final model. Based on 20, 000 simulated datasets with N = 311, the estimated power to detect the conditional indirect effect was 0.99, confirming that the achieved sample size provided adequate statistical power for the moderated mediation analysis.

### 2.3 Study participants

Inclusion criteria Participants were eligible if they met the following criteria: (1) diagnosed with ischemic stroke confirmed by cranial CT or MRI, in accordance with the Chinese Guidelines for the Diagnosis and Treatment of Acute Ischemic Stroke (2023) (23); (2) aged ≥18 years; (3) conscious and able to communicate effectively and complete the questionnaire independently; (4) voluntarily participated and provided written informed consent.

Exclusion criteria Participants were excluded if they: (1) had severe dysfunction of major organs (e.g., heart, liver, or kidney); (2) had malignant tumors, severe infections, or other serious diseases; (3) had cognitive impairment, psychiatric disorders, or consciousness disturbances preventing questionnaire completion; (4) had a history of psychiatric illness or were currently receiving psychiatric treatment; (5) provided incomplete questionnaires (missing >10% of items) or showed obvious response bias.

Patients with hemorrhagic stroke were not included in this study. This decision was based on substantial differences between hemorrhagic and ischemic stroke in pathophysiology, clinical course, recurrence patterns, and psychological responses, which may introduce additional heterogeneity in psychosocial outcomes. In addition, the study was conducted in the Department of Neurology of our hospital, where ischemic stroke is the predominant stroke subtype encountered in routine clinical practice. Therefore, restricting the sample to ischemic stroke patients helped ensure a more homogeneous study population and improved the internal validity of the findings.

### 2.4 Ethical considerations

This study was approved by the Ethics Committee of Deyang Hospital, Affiliated Hospital of Chengdu University of Traditional Chinese Medicine (Approval No.: 2025-04-011-KDD). Prior to the survey, participants were informed of the study purpose, procedures, and inclusion criteria. Written informed consent was obtained before participation. No personally identifiable information (e.g., names, home addresses, or contact details) was collected to ensure the confidentiality of participants’ health information.

### 2.5 Research instruments

#### 2.5.1 General information questionnaire

A self-designed questionnaire was used to collect sociodemographic and clinical data of patients with ischemic stroke, serving as control variables in subsequent analyses. The questionnaire covered ten dimensions, including age, marital status, education level, monthly disposable income, type of medical insurance, number of stroke episodes, disease duration, number of chronic conditions, and Modified Rankin Scale (mRS) score.

#### 2.5.2 Perceived recurrence risk scale

This scale was developed by Han et al. in 2022 (24) to assess patients’ perceived risk of stroke recurrence. The overall Cronbach’s α coefficient is 0.905. The scale consists of 25 items across four dimensions: warning symptoms (6 items), risk factors (7 items), health risks (6 items), and psychological and social risks (6 items). A 5-point Likert scale (1 = strongly disagree to 5 = strongly agree) is used, with total scores ranging from 25 to 125. Higher scores indicate higher levels of perceived recurrence risk. In this study, the Cronbach’s α coefficient was 0.822.

#### 2.5.3 Attitudes toward seeking professional psychological help scale–short form

The Chinese version of the ATSPPH-SF was used. Originally developed by Fischer et al. (1995) (25), the scale contains 10 items across two dimensions. The cumulative variance contribution of the two factors is 45.346%. The scale demonstrates acceptable reliability and validity in Chinese populations (26), with an overall Cronbach’s α of 0.82. Items are rated on a 4-point Likert scale (0 = disagree to 3 = agree), with total scores ranging from 0 to 30. Higher scores indicate more positive attitudes toward seeking professional psychological help.

#### 2.5.4 Hospital anxiety and depression scale

The HADS was developed by Zigmond and Snaith in 1983 (27) and is widely used to assess anxiety and depression in hospital settings (28). It consists of 14 items divided into two subscales: anxiety (HADS-A, 7 items) and depression (HADS-D, 7 items) (29). Each item is scored from 0 to 3, with subscale scores ranging from 0 to 21. Scores are categorized as follows: 0–7 (normal), 8–10 (mild), 11–14 (moderate), and 15–21 (severe). In this study, a score ≥8 on either subscale was considered indicative of anxiety and/or depression. In the present study, the Cronbach’s α coefficients were 0.898 for the HADS-Anxiety subscale and 0.792 for the HADS-Depression subscale, indicating acceptable to good internal consistency.

#### 2.5.5 Family APGAR index

The Family APGAR Index was developed by Smilkstein (30) to assess family functioning and has been widely applied in clinical settings (31). The Chinese version was introduced by Lü Fan and has demonstrated good reliability and feasibility (32). The scale includes five dimensions (adaptability, partnership, growth, affection, and resolve), with each item scored on a 3-point scale (0 = hardly ever, 1 = sometimes, 2 = almost always). Total scores range from 0 to 10, categorized as follows: 0–3 (severe dysfunction), 4–6 (moderate dysfunction), and 7–10 (good family functioning). In this study, the Cronbach’s α coefficient was 0.900.

### 2.6 Data collection and quality control

A standardized data collection procedure was implemented: (1)Investigator training: Five investigators with medical backgrounds received standardized training, including study objectives, questionnaire interpretation, communication skills, and ethical requirements. (2) Informed consent and questionnaire distribution: Eligible participants were recruited from neurology wards and rehabilitation outpatient clinics. Written informed consent was obtained prior to questionnaire administration. (3) Questionnaire completion: Participants completed questionnaires independently or via one-on-one interviews, depending on their physical condition. Investigators provided neutral assistance when necessary. (4) Data verification: Completed questionnaires were checked on-site for completeness and logical consistency. Questionnaires with <10% missing data were supplemented immediately; those with ≥10% missing data or significant inconsistencies were excluded and replaced.

### 2.7 Statistical analysis

All analyses were conducted using SPSS version 27.0 and the PROCESS macro (version 4.2) developed by Hayes. Descriptive statistics for categorical variables were presented as frequencies and percentages. Continuous variables were expressed as mean ± standard deviation (mean ± SD) after normality testing. Pearson correlation analysis was used to examine associations among key variables. Harman’s single-factor test was performed to assess common method bias. Mediation analysis was conducted using PROCESS Model 4, with ten control variables included to adjust for potential confounders. Moderated mediation analysis was performed using PROCESS Model 58 to examine the moderating effect of family care. The bootstrap method (5, 000 resamples) was used to estimate 95% confidence intervals (CIs) for mediation and moderation effects. Effects were considered statistically significant if the CI did not include zero, with a significance level of α = 0.05 (33). Further analyses were conducted to examine moderation effects at different levels of family care (mean −1 SD, mean, and mean +1 SD), and simple slope analyses were performed to illustrate the interaction effects.

## 3 Results

### 3.1 Common method bias test

Harman’s single-factor test was conducted to assess common method bias. The results showed that 15 factors had eigenvalues greater than 1, and the first factor accounted for 17.577% of the total variance, which was below the critical threshold of 40%. These findings indicate that no significant common method bias was present in this study.

### 3.2 General characteristics of participants

A total of 342 questionnaires were collected. After excluding 12 questionnaires that did not meet the inclusion criteria, 8 incomplete questionnaires, and 11 questionnaires with invalid responses (defined as straight-line responses, inconsistent answers on reverse-coded items, or implausibly short completion time), 311 valid questionnaires were included in the final analysis, yielding an effective response rate of 90.94%. Significant differences in negative emotions were observed across several sociodemographic and clinical variables, including age, marital status, type of medical insurance, monthly disposable income, number of stroke episodes, disease course, number of chronic diseases, and Modified Rankin Scale (mRS) score (all *p<0.05*; see **Table 1**). Overall, higher levels of negative emotions were more likely to be observed in patients with poorer socioeconomic status, greater disease burden, recurrent stroke, longer disease duration, multiple comorbidities, and more severe functional impairment. These findings suggest that negative emotions are not evenly distributed across stroke patients but tend to cluster in clinically vulnerable subgroups, highlighting the importance of considering baseline characteristics as control variables in subsequent analyses.

**Table 1:** Intergroup differences in negative emotions among patients with different sociodemographic characteristics (N = 311).

Variables	Category	N	%	Negative emotions	T/F	P
Gender	Male	162	52.09	23.91 ± 5.27	0.224	0.636
	Female	149	47.91	24.20 ± 5.45		
Age(years)	<45	27	8.68	27.04 ± 5.00	6.583	0.000
	45~60	84	27.01	25.26 ± 4.90		
	60~75	152	48.87	23.05 ± 5.29		
	>75	48	15.43	23.44 ± 5.57		
Marital status	Married	208	66.88	23.51 ± 4.92	3.644	0.027
	Unmarried/Divorced	78	21.05	25.40 ± 5.53		
	Widowed	25	8.04	24.36 ± 7.36		
Education level	Primary school and below	77	24.76	24.23 ± 5.45	0.627	0.598
	Junior high school	87	27.97	24.54 ± 5.46		
	Senior high school	88	28.30	23.47 ± 5.27		
	College degree and above	59	18.97	23.97 ± 5.20		
Medical insurance type	Urban and rural residents medical insurance	162	52.09	24.53 ± 5.17	7.263	0.000
	Urban employee medical insurance	120	38.59	22.81 ± 5.31		
	Others	29	9.32	26.52 ± 5.37		
Monthly disposable income(RMB)	<1000	52	16.72	25.87 ± 4.91	3.889	0.009
	1000~2999	169	54.34	23.74 ± 5.54		
	3000~5999	61	19.61	24.38 ± 5.20		
	≥6000	29	9.32	21.93 ± 4.36		
Number of stroke attacks	First onset	182	58.52	23.23 ± 5.31	10.663	0.001
	Recurrence	129	41.48	25.21 ± 5.20		
Disease course (months)	Acute phase (≤1 month)	43	13.83	22.05 ± 3.91	8.359	0.000
	Recovery phase (1~6 months)	102	32.80	23.13 ± 4.96		
	Sequelae phase (>6 months)	166	53.38	25.14 ± 5.66		
Number of chronic diseases	0	37	11.90	21.65 ± 5.94	4.820	0.003
	1	87	27.97	23.89 ± 5.42		
	2	133	42.77	24.08 ± 5.11		
	>3	54	17.36	25.89 ± 4.81		
Modified Rankin Scale score	0~2	175	56.27	21.89 ± 5.08	42.094	0.000
	3~4	93	29.90	26.55 ± 4.32		
	5~6	43	13.83	27.47 ± 4.25		

### 3.3 Correlation analysis among variables

**Figure 2:**
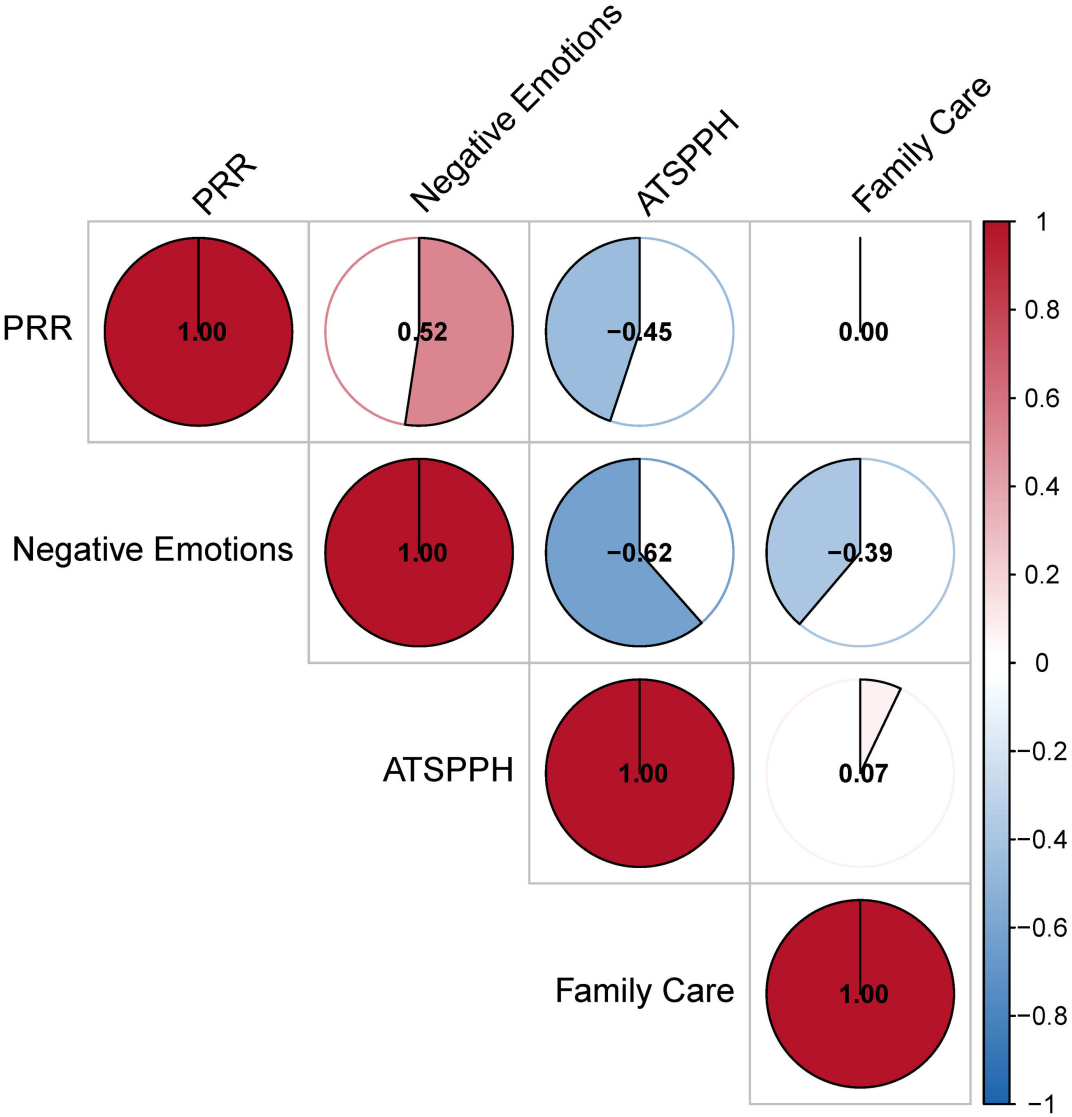
Thermodynamic diagram of correlation between core variables. PRR, Perception of Recurrence Risk; ATSPPH, Attitudes toward seeking professional psychological help.

After testing for normality, continuous variables were expressed as mean ± standard deviation. The mean scores were as follows: perceived recurrence risk (84.40 ± 8.71), negative emotions (24.05 ± 5.35), attitudes toward seeking professional psychological help (14.52 ± 3.86), and family care (6.57 ± 1.80). Spearman correlation analysis revealed that perceived recurrence risk was positively correlated with negative emotions (*r=0.524, p<0.01*). Perceived recurrence risk was negatively correlated with attitudes toward seeking professional psychological help (*r=−0.449, p<0.01*). Negative emotions were negatively correlated with both attitudes toward seeking professional psychological help (*r=−0.615, p<0.01*) and family care (*r=−0.388, p < 0.01*). Detailed results are presented in **Figure 2**.

### 3.4 Main effects analysis

After controlling for demographic variables (e.g., age, gender, and education level), hierarchical regression analysis showed that perceived recurrence risk was significantly positively associated with negative emotions (β = 0.350, p < 0.001). The model explained 46.3% of the variance in negative emotions. These findings indicate that patients with higher perceived recurrence risk are more likely to experience negative emotions, supporting Hypothesis 1 (see **Table 2**).

**Table 2:** Hierarchical regression analysis of perception of recurrence risk and negative emotions.

Variables	Model 1				Model 2			
	β	SE	P	VIF	β	SE	P	VIF
Age	-0.186	0.312	< 0.001	1.138	-0.91	0.304	0.055	1.246
Gender	0.013	0.495	0.779	1.037	0.026	0.462	0.553	1.039
Marital status	-0.147	0.449	0.002	1.032	-0.078	0.430	0.078	1.089
Education level	-0.051	0.310	0.403	1.827	-0.017	0.290	0.769	1.841
Monthly disposable income	-0.122	0.322	0.016	1.211	-0.143	0.300	0.002	1.216
Number of stroke attacks	0.087	0.539	0.081	1.199	0.078	0.503	0.096	1.200
Disease course	-0.116	0.350	0.014	1.071	-0.076	0.329	0.085	1.090
Number of chronic diseases	0.164	0.354	0.006	1.729	0.202	0.332	< 0.001	1.746
MRS	0.523	0.410	< 0.001	1.486	0.360	0.421	< 0.001	1.806
Medical insurance type	-0.227	0.460	< 0.001	1.547	-0.180	0.432	< 0.001	1.574
Perception of Recurrence Risk	-	-	-	-	0.350	0.032	< 0.001	1.480
R2	0.380				0.463			
△R2	0.359				0.443			
F	18.366***				46.161***			

^*^p < 0.05, ^**^p < 0.01, ^***^p < 0.001.

### 3.5 Mediating effect of attitudes toward seeking professional psychological help

The mediating effect was tested using PROCESS Model 4. As shown in **Table 3**, perceived recurrence risk had a significant direct effect on negative emotions (*B=0.191, p<0.001; 95% CI: 0.134–0.247*). In addition, the indirect effect through attitudes toward seeking professional psychological help was also significant (*B=0.131, p<0.001; 95% CI: 0.082–0.192*), accounting for 40.68% of the total effect. These results indicate that attitudes toward seeking professional psychological help partially mediate the relationship between perceived recurrence risk and negative emotions, supporting Hypothesis 2.

**Table 3:** Mediating effect of psychological help-seeking attitude between perception of recurrence risk and negative emotions.

Effect type	Effect value	SE	95%CI	P	Relative effect value (%)
Direct effec	0.191	0.029	0.134~0.247	0.000	59.32%
Indirect effect	0.131	0.028	0.082~0.192	0.000	40.68%
Total effect	0.322	0.030	0.263~0.380	0.000	100.00%

### 3.6 Moderating effect of family care

**Figure 3:**
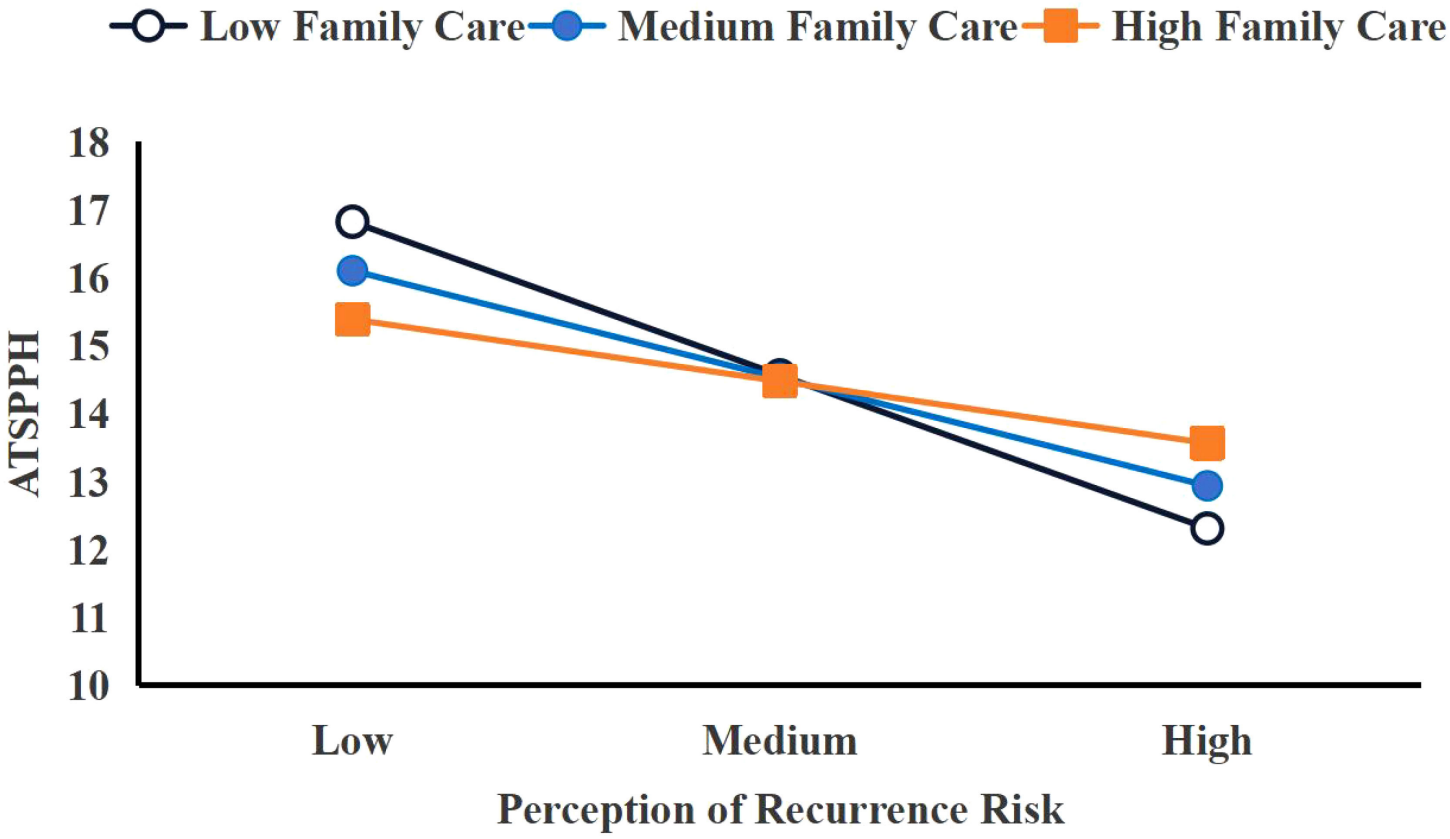
Simple slope plot of the moderating effect of family care on the association between perceived recurrence risk and ATSPPH.

The moderating effect of family care was examined using PROCESS Model 58. For the first stage of the model, where perceived recurrence risk predicted attitudes toward seeking professional psychological help, the interaction between perceived recurrence risk and family care was significantly associated with help-seeking attitudes (*β = 0.043, p = 0.001, 95% CI: 0.017–0.069; see* **Table 4**). Simple slope analysis (**Figure 3**) showed that the negative association between perceived recurrence risk and help-seeking attitudes was attenuated as family care increased, with the simple slope changing from −0.259 (*at mean −1 SD = 4.767*) to −0.105 (*at mean +1 SD = 8.371*). The Johnson–Neyman test identified a significance threshold of 8.890, indicating that the negative association remained statistically significant when family care was below this value. Overall, these findings suggest that higher levels of family care weaken the negative association between perceived recurrence risk and help-seeking attitudes.

**Table 4:** Analysis and test of the moderating effect of family care.

Variable	ATSPPH			Negative emotions		
	β	SE	T	β	SE	T
Perception of Recurrence Risk	-0.463	0.084	-5.542***	0.208	0.025	8.188***
Family Care	-3.642	1.161	-3.138**	0.008	0.411	0.020
Perception of Recurrence Risk×Family Care	0.043	0.013	3.282***	-	-	-
ATSPPH	-	-	-	-0.162	0.178	-0.909
ATSPPH×Family Care	-	-	-	-0.075	0.028	-2.679**
R2	0.233			0.590		
F	31.082***			109.968***		

^*^p < 0.05, ^**^p < 0.01, ^***^p < 0.001.

**Figure 4:**
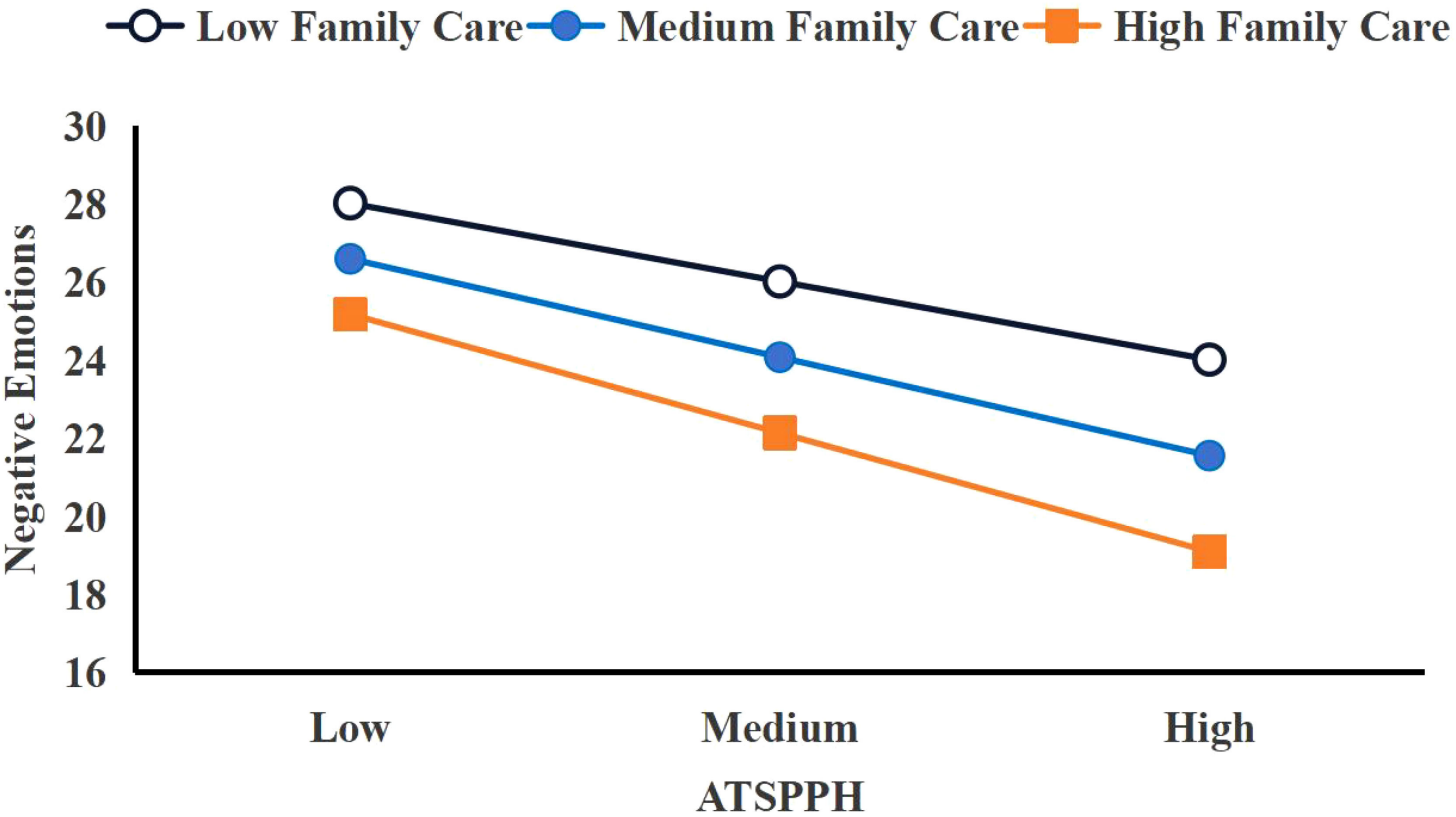
Simple slope plot of the moderating effect of family care on the association between ATSPPH and negative emotions.

The interaction between attitudes toward seeking professional psychological help and family care was significantly associated with negative emotions (*β = −0.075, p = 0.008, 95% CI: −0.130 to −0.020*). Simple slope analysis (**Figure 4**) showed that as family care increased from 4.767 to 8.371, the negative association between help-seeking attitudes and negative emotions became stronger (*from −0.519 to −0.788*). The Johnson–Neyman test indicated that this association remained statistically significant across the entire observed range of family care. These findings suggest that family care may play a role in moderating the association between help-seeking attitudes and negative emotions. Specifically, higher levels of family care may be associated with a more pronounced negative relationship between help-seeking attitudes and negative emotions, whereas lower levels of family care may be associated with a weaker relationship. These results are consistent with Hypotheses 3 and 4.

For the second stage of the model (help-seeking attitudes and negative emotions), the interaction between help-seeking attitudes and family care was significantly associated with negative emotions (*β = −0.075, p = 0.008, 95% CI: −0.130 to −0.020*). Simple slope analysis showed that the negative association between help-seeking attitudes and negative emotions became stronger as family care increased, with the slope changing from −0.519 to −0.788 across levels of family care (4.767 to 8.371). The Johnson–Neyman analysis indicated that this association remained statistically significant across the entire observed range of family care.

In addition, the conditional indirect effect analysis indicated that the mediating effect of help-seeking attitudes was significant at low, mean, and high levels of family care (95% CIs did not include zero). The magnitude of the indirect effect decreased with increasing family care, from 0.134 to 0.082 (**Table 5**), suggesting variation in the strength of the indirect association across levels of family care.

**Table 5:** The impact of different levels of adjustment on the mediation effect size in the mediation model.

Level of family care	Mediating effect size	SE	BootLLCI	BootULCI
-1SD	0.134	0.038	0.062	0.209
Mean	0.119	0.026	0.073	0.177
+1SD	0.082	0.039	0.017	0.169

These findings suggest that family care is associated with differences in both the direct and indirect statistical relationships among study variables, providing partial support for Hypotheses 3 and 4.

## 4 Discussion

### 4.1 Descriptive statistics and correlations among study variables

This study found that patients with ischemic stroke exhibited a moderately high level of perceived recurrence risk, relatively high levels of negative emotions (23.91 ± 5.45), generally negative attitudes toward seeking professional psychological help, and a moderate level of family care with considerable individual variability. These findings are consistent with recent studies (6).

The elevated perceived recurrence risk is closely related to the characteristics of stroke, including high recurrence, disability, and mortality rates. Patients often experience persistent fear of recurrence, forming a chronic psychological threat (34). This perception represents a realistic stress response to disease burden rather than excessive worry and serves as a key source of negative emotions.

The high prevalence of negative emotions confirms that post-stroke anxiety and depression have become the second most common complication after physical dysfunction (9). These emotional disturbances not only reduce treatment adherence but may also exacerbate physiological dysfunction through neuroendocrine mechanisms, such as increased blood pressure and thrombosis risk, forming a vicious cycle between emotional deterioration and physical impairment (35).

The generally negative attitudes toward seeking professional psychological help reflect the current challenges in stroke-related mental health services in China. Patients often experience stigma and may perceive psychological problems as a sign of weakness (36). In addition, limited availability of mental health services in primary care settings, insufficient family awareness, and restricted insurance coverage further hinder access to psychological support (37).

Family care was found to be moderate overall but highly heterogeneous, suggesting that families can function both as protective resources (38) and as potential stressors (39). Inadequate support, poor communication, and caregiver burden may increase patients’ feelings of loneliness and self-negation, thereby weakening psychological adaptation.

Collectively, these findings indicate that stroke rehabilitation has entered a new stage characterized by integrated physical and psychological care, family involvement, and continuous management. Solely focusing on physical rehabilitation is insufficient; routine psychological screening and intervention should be incorporated into standard clinical practice.

### 4.2 Mediating role of attitudes toward seeking professional psychological help

From the perspective of Stress and Coping Theory (15), perceived recurrence risk reflects a threat-related cognitive appraisal. When patients perceive recurrence as highly threatening and uncontrollable, stress-related psychological responses may be activated and are associated with higher levels of negative emotions (6, 40). Attitudes toward seeking professional psychological help, as an important coping orientation, may serve as a potential pathway linking stress appraisal and emotional outcomes (41). Specifically, higher perceived recurrence risk may be associated with avoidance-oriented coping tendencies and reduced willingness to seek professional psychological support, which may limit access to external psychological resources and emotional support, thereby being associated with more severe negative emotions. In contrast, more positive help-seeking attitudes may be associated with more adaptive coping patterns and greater utilization of psychological support resources, which may help alleviate psychological distress.

From a clinical perspective, current interventions addressing recurrence risk perception and help-seeking attitudes remain limited. Patients often lack accurate information regarding recurrence risk, which may contribute to exaggerated perceptions of uncertainty (42), while structured risk communication in clinical practice is still insufficient. In addition, stigma and limited accessibility of psychological services may further hinder help-seeking behaviors (43). These findings suggest that improving psychological outcomes in stroke patients may benefit from a dual-focus approach targeting both risk perception and help-seeking attitudes. Interventions may consider shifting from fear-based education toward supportive and empowerment-oriented communication, while also reducing stigma and improving access to psychological services, particularly for high-risk groups (44, 45).

### 4.3 Moderating role of family care

This study demonstrated that family care exerted significant moderating effects on both stages of the mediation pathway, forming a moderated mediation model with moderation at both stages, thereby supporting Hypotheses 3 and 4. In the first stage, higher family care attenuated the negative association between perceived recurrence risk and help-seeking attitudes. Adequate family support may reduce perceived stigma, enhance psychological safety, and facilitate patients’ willingness to seek professional help. In contrast, insufficient family care may be associated with greater feelings of isolation and a tendency toward avoidant coping (46). In the second stage, higher family care strengthened the negative association between help-seeking attitudes and negative emotions, suggesting a potential synergistic effect between family support and professional psychological resources. Conversely, low family care may weaken the sustained benefits of help-seeking behaviors (47).

These findings are consistent with Social Support Theory, which emphasizes the buffering role of family support as a key protective resource. Given that stroke rehabilitation in China is largely home-based, family support plays a particularly important role in patients’ psychological adjustment (48). However, family members may have limited knowledge of psychological care, which can lead to overprotection or negative communication patterns and may inadvertently worsen patients’ emotional states. Importantly, these results suggest that family care is not merely companionship, but also involves effective communication, emotional support, and facilitation of professional psychological help-seeking. Its buffering role appears particularly relevant for high-risk patients, providing empirical support for the development of family-centered psychological interventions.

## 5 Practical implications

The findings of this study suggest several clinically relevant implications for ischemic stroke rehabilitation. First, consistent with previous research highlighting the importance of risk perception in post-stroke psychological adaptation (3), stratified screening may be considered to identify high-risk subgroups (e.g., patients with recurrent stroke, severe disability, or low socioeconomic status) for early psychological support. Second, in line with evidence that excessive recurrence fear is associated with emotional distress and maladaptive coping (49), recurrence risk communication may benefit from shifting away from fear-based messaging toward more supportive and controllable framing, emphasizing preventability and self-management strategies. Third, consistent with studies demonstrating that stigma and low help-seeking attitudes are associated with reduced utilization of psychological services in stroke populations (50), interventions aimed at reducing stigma and improving accessibility of mental health services may be warranted. Fourth, echoing prior findings on the buffering role of family functioning in stroke outcomes (51), family-centered interventions may be considered, with emphasis on improving communication, emotional support, and facilitation of professional psychological help-seeking. Finally, a staged emotional management approach across acute, rehabilitation, and chronic phases may help ensure continuity of psychological care.

## 6 Limitations

Several limitations should be acknowledged when interpreting the findings of this study. First, due to the cross-sectional design, causal inferences cannot be established, and the observed associations among perceived recurrence risk, help-seeking attitudes, family care, and negative emotions should be interpreted with caution, consistent with previous psychosocial research in stroke populations emphasizing the need for longitudinal designs to clarify temporal mechanisms. Second, all data were obtained through self-reported questionnaires, which may introduce recall bias and social desirability bias, a common limitation in studies of psychological outcomes among patients with stroke. Third, the sample was recruited from a limited number of hospitals in Sichuan Province, which may restrict the generalizability of the findings due to potential regional differences in healthcare resources and family support systems. In addition, this study included only patients with ischemic stroke, excluding those with hemorrhagic stroke because of substantial differences in pathophysiology, clinical course, and psychological response patterns; therefore, the applicability of the proposed model to other stroke subtypes requires further validation. Finally, although several covariates were controlled, unmeasured factors such as broader social support networks, rehabilitation intensity, and psychological resilience were not included, which may also influence emotional outcomes. Future longitudinal, multicenter studies incorporating additional psychosocial variables are needed to further validate and extend the present findings.

## 7 Conclusion

In conclusion, this study demonstrates that perceived recurrence risk is associated with negative emotions in patients with ischemic stroke, both directly and indirectly through help-seeking attitudes, while family care plays a dual moderating role. These findings support the importance of integrating psychological coping processes and family support into stroke rehabilitation. However, causal interpretations should be made cautiously due to the cross-sectional design, and future longitudinal research is needed to further clarify these relationships.

## Data Availability

The original contributions presented in the study are included in the article/supplementary material. Further inquiries can be directed to the corresponding author.
